# LBP reduces theinflammatory injuryof kidney in septic rat and regulates the Keap1-Nrf2∕ARE signaling pathway^1^

**DOI:** 10.1590/s0102-865020190010000003

**Published:** 2019-02-14

**Authors:** Yayi Huang, Fang Zhou, Chen Shen, Huaxin Wang, Yeda Xiao

**Affiliations:** I PhD, Department of Anesthesiology, Renmin Hospital of Wuhan University, China. Conception and design of the study, manuscript preparation and writing.; II Graduate student, Department of Anesthesiology, Renmin Hospital of Wuhan University, China. Acquisition of data, technical procedures.; III PhD, Department of Anesthesiology, Renmin Hospital of Wuhan University, China. Technical procedures, final approval.

**Keywords:** Antioxidant Response Elements, Lycium, Kelch-Like ECH-Associated Protein 1, Rats

## Abstract

**Purpose:**

To investigate the influence of lycium barbarum polysaccharides (LBP), a functional derivative from lycium barbarum, on septic kidney injury.

**Methods:**

The SD male rats were randomly divided into 8 groups. The concentration of IL-1β, IL-6, IL-8, TNF-α, NF-κB and ROS, in kidney cortex homogenates after 12 h treatments were determined by enzyme-linked immunosorbent assay and ROS test kit, respectively. Morphology observation of kidney tissue was conducted with HE staining. The mRNA and protein expression levels of Nrf2, HO-1, NQO1, NF-κB, and Keap1 in kidney tissues were determined by qRT-PCR and Western blot, respectively.

**Results:**

LPS treatment significantly increased the oxidative stress. After LBP treatment, the ROS content reduced significantly in a dose-depend manner. However, the levels of HO-1, NQO1 and Nrf2 as molecular elements that respond to oxidative stress were further increased. Also, administration of LBP increased the levels of NF-κB and Keap1, and decreased the levels of Nrf2 in the Keap 1-Nrf2∕ARE signaling pathway. By administrating the brusatol, the inhibition of Nrf2 enhanced the expression of NF-κB, inhibits the antioxidant responses, and further reverse the protective effect of LBP on the LPS induced septic kidney injury.

**Conclusion:**

Lycium barbarum polysaccharides can reduce inflammation and activate the antioxidant responses via regulating the level of pro-inflammatory cytokines and the Keap1-Nrf2/ARE signaling pathway.

## Introduction

 Sepsis is a kind of systemic inflammatory response syndrome (SIRS), mainly infected by bacteria, fungi, viruses and parasites^1^. The SIRS caused by sepsis may incur function damage of systemic multiple organ, like septic acute kidney injury (AKI)^2^. Nuclear factor-erythroid 2 related factor 2 (Nrf2) is an important transcription factor in oxidative stress response. It regulates antioxidant response elements (AREs)-mediate depression of antioxidant enzyme and cytoprotective proteins. Moreover, multiple studies have demonstrated that Nrf2-Keap1 (Kelch-like ECH-associated protein 1)-ARE signal pathway involves in the endogenous antioxidant defense mechanisms and it is an important signaling pathway in oxidative stress system^3^. 

 Lycium barbarum polysaccharides (LBPs) are functional derivative from Lycium barbarum. The therapeutic effects of LBP on human health has been explored in the past years. For example, LBPs have been reported to inhibit cell proliferation, induce apoptosis, and interrupt intracellular calcium balance of cancer cells^4^. Also, it has been demonstrated that LBPs exhibit anti-aging and neuroprotective functions both in vitro and in vivo^5^ ^,^ ^6^ In addition, as a potent antioxidant, LBPs have been shown to up-regulate the level of antioxidant biomarkers in human serum^7^ and protect the body from chemical/exercise-induced oxidative stress^8^ ^,^ ^9^. Recently, Kawara *et al*.^10^ reported that shikonin a major component of zicao (a Chinese herbal medicine with various biological activities) possesses antioxidant effects via activation of Nrf2 against lipopolysaccharide (LPS)-induced AKI in a murine model. However, whether LBP has protective effects on inflammatory injury and oxidative stress reaction of septic rat kidney is still unclear. 

 In this study, we found the protective effect of LBP treatment in septic rat kidney. LBP can up-regulate the level of Nrf2 and pro-inflammatory cytokines, and further regulating the Keap1-Nrf2/ARE signaling pathway, consequently activating the antioxidant responses and reducing inflammation.

## Methods

###  Sepsis animal model 

 All experiments were approved by the Animal Care and Use Committee of Renmin Hospital of Wuhan University following the Principles of Laboratory Animal Care (NIH publication no. 85-23, revised 1985).

 SD male rats (weight 220-240 g; n=48; 6 in each group) recruited in this study were obtained from Shanghai Laboratory Animal Center. After feeding one week, the rats were randomly divided into 8 groups: (1) Normal control group (Con) : normal feeding; (2) LPS model group (LPS): intraperitoneal injection with LPS (5 mg/kg); (3) Ulinastatin group (ULI): intravenous injection with Ulinastatin (10000 U/kg); (4) Low LBP dose group (LBP-1): giving intragastric administration with 200 mg/kg LBP 1 h after LPS injection; (5) Middle LBP dose group (LBP-2): giving intragastric administration with 400 mg/kg LBP 1 h after LPS injection; (6) High LBP dose group (LBP-3): giving intragastric administration with 800 mg/kg LBP 1 h after LPS injection; (7) Brusatol group: intraperitoneal injection with LPS (800 mg/kg) together with 2 mg/kg of brusatol, then LBP was administrated 1 h after; (8) DMSO control group (DMSO): intraperitoneal injection with DMSO. 

###  Specimen collection 

 The experimental rats after 12h of treatments were anaesthetized by intraperitoneal injection with 7% chloral hydrate. Anesthesia rats were then fixed on the operating table in supine position. Unilateral kidneys of the SD rats were separated to fix with formalin,then gradient alcohol dehydration and embedding. The other side of kidney were used for the preparation of tissue homogenate and the extraction of protein and RNA. Also, blood was collected and heparinized, and then blood urea nitrogen (BUN) and creatinine were analyzed using Reflotron Plus Clinical Chemistry Analyzer (Roche, USA). 

###  Enzyme-linked immunosorbent assay (ELISA) 

 Inflammatory cytokines (IL-1β, IL-6, IL-8, TNF-α and NF-κB) were determined in kidney cortex homogenates after 12 h treatments by using specific kits (Invitrogen, USA) according to the manufacturer’s instructions. 

###  Determination the ROS content in kidney tissues 

 ROS test kit (Beyotime, China) was used to determinate the ROS content in kidney cortex homogenates. ROS formation was quantified and expressed as pmol DCF/min/mg protein.

###  HE staining and morphology observation of kidney tissue 

 The unilateral kidney sections of the SD rats were stained with HE (hematoxylin-eosin) staining. The pathological changes of kidney tissue were observed under optical microscope. The kidney injury scores were determined by light microscopy on a scale of 0-5: 0 = normal histology; 1 = degeneration only without necrosis; and 2 (< 25%), 3 (< 50%), 4 (< 75%), and 5 (> 75%) = necrosis, degeneration, regeneration, tubular dilatation, protein casts, and interstitial lymphocytic infiltration levels of the proximal convoluted tubules.

###  Immunohistochemical study 

 The unilateral kidney sections were incubated with anti-Nrf2 monoclonal antibody (1 : 100; Santa Cruz, USA), followed by the incubation with goat anti-rabbit IgG secondary antibody (Santa Cruz, USA). The immune reaction resulted in the oxidation of the 3,3′-diaminobenzidine by peroxidase (DAKO, USA) into an insoluble brown precipitate. The positive cells were visualized as a brown staining.

###  Quantitative real-time reverse transcription-PCR (qRT-PCR) 

 Total RNA of kidney tissue in SD rats was extracted using the TRIzol reagent (Takara, China), according to the manufacturer’s instructions. cDNA was synthesized from 1 μg of total RNA using PrimeScript™ RT Master Mix (Takara, China). RT-PCR was performed in a CFX96^TM^ real-time detection system (ABI Prism 7500) with a total volume of 20 μL, containing 10 μL 2 × TransStart Tip Green qPCR Super Mix (Trans), 1 μL of each primer, 1 μL cDNA templates and 7 μL ddH_2_O. The specific primer sequences are listed in Table 1. The amplification conditions are as follows: initial denaturation at 94°C for 30s; followed by 40 cycles of denaturation at 94°C for 5s, annealing at 56°C for 15s; and a final extension at 72°C for 10s. The relative expression level was determined using the 2^−ΔΔCt^ method. Each treatment was repeated in triplicates. The data were presented as the means ± standard error (S.E).

**Table 1:** Primer sequences of the specific genes.

Target genes	Sequences (5′→3′)	
HO-1	Forward: CTGGAAGAGGAGATAGAGC	Reverse: CTGGTGTGTAAGGGATGG
NQO1	Forward: AACGACATCACAGGGGAG	Reverse: GCACCCCAAACCAATACA
NF-κB	Forward: ACGATCTGTTTCCCCTCATCT	Reverse: TGCTTCTCTCCCCAGGAATA
Keap1	Forward: TGCTCAACCGCTTGCTGTATG	Reverse: CCAAGTGCTTCAGCAGGTACA
Nrf2	Forward: GCCAGCTGAACTCCTTAGAC	Reverse: GATTCGTGCACAGCAGCA
GAPDH	Forward: TTCAATGGCACAGTCAAGGC	Reverse: TCACCCCATTTGATGTTAGCG

###  Western blot analysis 

 The total proteins from kidney tissue were extracted using the Cytoplasmic and Nuclear Protein Extraction Kit (ProMab, USA), and the the protein concentrations were determined using the BCA method (Thermo Fisher Scientific, USA). Then, protein samples were subjected to SDS-PAGE and transferred to a polyvinylidene difluoride (PVDF) membrane. After blocking with PBST (PBS containing 0.1% Tween-20) containing 5% non-fat milk, the membranes were washed for 3 times with PBST, and then incubated with rabbit anti Nrf2, HO-1, NQO1, NF-κB, Keap1 of ratantibodies (1:500 dilution; Beyotime, China) for 1 h at room temperature (RT). After 3 more times of washing with PBST, the membranes were incubated with goat anti-rabbit IgG (1:2000 dilution; Beyotime, China) for 1 h at RT. The immunoblot signal was detected using Pierce ECL Western Blotting Substrate (Thermo Fisher Scientific, USA).

###  Statistical analysis 

 Data were compared by one-way analysis of variance (ANOVA), followed by Tukey’s test with SPSS 18.0statistical software. All data were represented as the mean ± S.E. Differences were considered significant when P<0.05.

## Results 

###  Influence of LBP on the expressions of immune factors in sepsis rat kidney 

**Figure 1:**
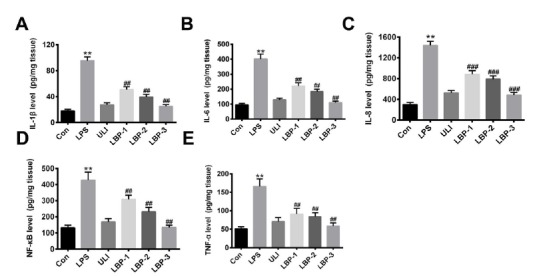
Influence of LBP on the expressions of immune factors in sepsis induced rat kidney. (**A**) IL-1β; (**B**) IL-6; (**C**) IL-8; (**D**) NF-κB; (**E**) TNF-α. **, P<0.01, *versus* the control group; ##, P<0.01, *versus* the LPS group. Normal control group (Con): normal feeding; LPS model group (LPS): intraperitoneal injection with LPS (5 mg/kg); Ulinastatin group (ULI): intravenous injection with Ulinastatin (10000 U/kg); LBP-1 group: giving intragastric administration with 200 mg/kg LBP 1h after LPS injection; LBP-2 group: giving intragastric administration with 400 mg/kg LBP 1h after LPS injection; LBP-3 group: giving intragastric administration with 800 mg/kg LBP 1h after LPS injection.

 To investigate the inflammatory response of the LPS induced sepsis rat to LBP treatment, inflammatory cytokines (IL-1β, IL-6, IL-8, TNF-α and NF-κB) were determined in kidney cortex homogenates after 12h treatment through the Enzyme-Linked Immunosorbent Assay (ELISA). The result (Fig. 1 A-E) showed that the concentration of IL-1β, IL-6, IL-8, TNF-α and NF-κB increased significantly after injection with LPS, compared with the control (P<0.01). Ulinastatin is a serine protease inhibitor, which can decrease inflammatory cytokine levels and mortality in experimental sepsis^11^. Therefore, we further conduct intravenous administration of ulinastatin to the rat. The result showed that the expression level of all the five cytokines significantly decreased (P<0.01, *versus* the LPS group). Consistently, after different doses of LBP injection, the expression levels of IL-1β, IL-6, IL-8, TNF-α and NF-κB presented a significant reduction (P<0.01, *versus* the LPS group) in a concentration depended manner. All these results indicate that the LBP exerts a protective effect on the kidney of the sepsis induced rat.

###  LBP has a protective role against LPS induced septic kidney injury 

**Figure 2:**
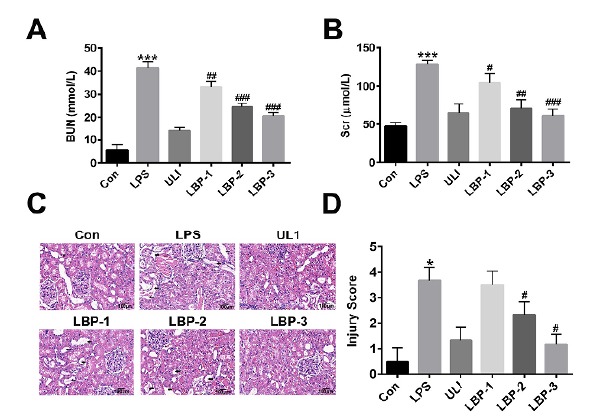
Function and Pathological morphology observation of kidney tissue among groups. (**A**) Blood urea nitrogen (BUN) and (**B**) creatinine levels in heparinized rat blood samples. (**C**) Pathological morphology observation of kidney tissue. (**D**) The kidney injury scores determined by light microscopy on a scale of 0-5. **, P<0.01, *versus* the control group; ##, P<0.01, *versus* the LPS group. Normal control group (Con): normal feeding; LPS model group (LPS): intraperitoneal injection with LPS (5 mg/kg); Ulinastatin group (ULI): intravenous injection with Ulinastatin (10000 U/kg); LBP-1 group: giving intragastric administration with 200 mg/kg LBP 1h after LPS injection; LBP-2 group: giving intragastric administration with 400 mg/kg LBP 1h after LPS injection; LBP-3 group: giving intragastric administration with 800 mg/kg LBP 1h after LPS injection.

 In order to understand the functional and pathological changes of kidney tissue after LBP intervention, the serum BUN and Cr were analyzed at the end of the treatment period, and HE staining was utilized to observed the unilateral kidney sections of the SD rats after 12h of intervention. As shown in Figure 2 (A,B), the concentration of BUN and creatinine raised dramatically after LPS treatment (P<0.001, versus the control group), indicating that kidney function was declined. Consistently, our HE staining results (Fig. 2 C,D) showed that, in the control group, normal organization structure was observed in rat kidney tissue and there were no obvious abnormal changes. However, lots of inflammation cells aggregation and cellular swelling and infiltration were observed in the kidney tissue after 12h post-injection of LPS. As expected, in the LBP intervention groups, the concentration of BUN and creatinine reduced significantly (P<0.05, *versus* the LPS group) in a concentration dependent manner (Fig. 2 A,B). Also, in the HE staining results, cellular edema, structural disorder, and inflammatory cell infiltration can still be observed, nevertheless much less than the LPS group (Fig. 2 C,D). These results indicate that administration of LBP could improve kidney tissue injury of septic rats.

###  Effect of LBP on the antioxidant response in LPS induced septic kidney 

**Figure 3:**
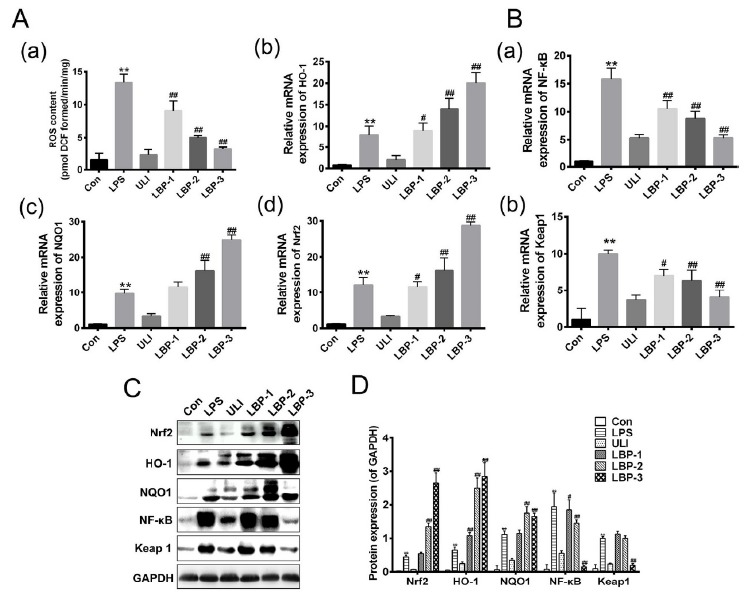
LBP treatment protects the septic kidney injury by activating the antioxidant responses through Keap 1- Nrf2∕ARE signaling pathway. (**A**) Effect of LBP on the antioxidant response in LPS induced septic kidney. (**B**) Effect of LBP on the Keap 1- Nrf2∕ARE signaling pathway related genes. (**C**) Western blot analysis of antioxidant responses and Keap 1- Nrf2∕ARE signaling pathway related proteins. (D) Quantitative analysis of the Western blot results. **, P<0.01, *versus* the control group; ##, P<0.01, versus the LPS group. Normal control group (Con): normal feeding; LPS model group (LPS): intraperitoneal injection with LPS (5 mg/kg); Ulinastatin group (ULI): intravenous injection with Ulinastatin (10000 U/kg); LBP-1 group: giving intragastric administration with 200 mg/kg LBP 1h after LPS injection; LBP-2 group: giving intragastric administration with 400 mg/kg LBP 1h after LPS injection; LBP-3 group: giving intragastric administration with 800 mg/kg LBP 1h after LPS injection.

 To evaluate the role of LBP on the oxidative stress, we evaluated the content of ROS in kidney homogenates. The result showed that the content of ROS in LPS group increased significantly compared with the control. After different doses of LBP treatments, the ROS content reduced significantly in a dose-depend manner (Fig. 3A-a). To further confirm the role of LBP on antioxidant response, we evaluated the mRNA and protein expressions of HO-1, NQO1, Nrf2 as molecular elements that respond to oxidative stress. The result showed that compared with the control group, the mRNA (Fig. 3A) and protein (Fig. 3 C,D) expression levels of HO-1, NQO1, Nrf2 after administration of LPS was increased (P<0.01). After LBP intervention, the expression levels of HO-1, NQO1 and Nrf2 were further increased with the increase of LBP doses (P<0.01 when treated with 400 mg/kg and 800 mg/kg LBP, *versus* the LPS group). These results indicate that LBP treatment can protect the septic kidney injury by activating the antioxidant responses.

###  The effect of LBP on the Keap1- Nrf2∕ARE signaling pathway 

 To further investigate the effect mechanism of LBP on the Keap1-Nrf2∕ARE signaling pathway, qRT-PCR and western blot were utilized to determinate the expression level of NF-κB and Keap1 in kidney homogenates among groups. The qRT-PCR results showed that compared with the control group, the mRNA level of NF-κB and Keap1 after injection LPS significantly increased (P<0.01), while LBP treatment abolished these increases in a dose-dependent manner (Fig. 3B). Consistent results were also observed by the Western blot (Fig. 3 C,D).

###  LBP treatment increases the Nrf2 accumulation in nucleus by activating Keap1-Nrf2∕ARE signaling pathway 

**Figure 4:**
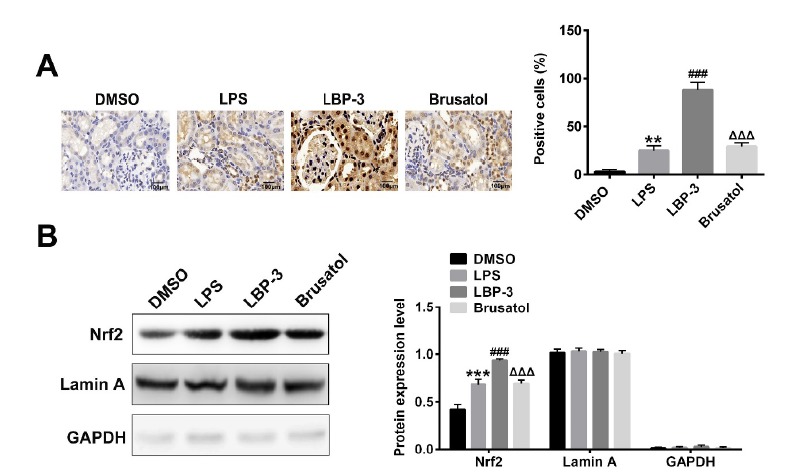
LBP treatment increases the Nrf2 accumulation in nucleus by activating Keap1- Nrf2∕ARE signaling pathway. (**A**) Immunohistochemical study of Nrf2 positive cells in kidney tissue after different treatments. (**B**) Nuclear accumulation of Nrf2 among different groups determined by Western blot. ***, P<0.001, versus the DMSO group; ###, P<0.001, *versus* the LPS group; ΔΔΔ, P<0.001, *versus* the LBP group. DMSO control group (DMSO): intraperitoneal injection with DMSO. LPS model group (LPS): intraperitoneal injection with LPS (5 mg/kg); LBP-3 group: giving intragastric administration with 800 mg/kg LBP 1h after LPS injection; Brusatol group: intraperitoneal injection with LPS together with 2 mg/kg of brusatol, then LBP was administrated 1h after.

 In order to further confirm the role of LBP on the Keap1- Nrf2∕ARE signaling pathway, the brusatol, an inhibitor of Nrf2, was applied in this study. As shown in Figure 4A, the immunohistochemical study demonstrated that the LBP treatment could dramatically increase the percentage of Nrf2 positive cells when compared with the LPS group (P<0.001), which can be successfully reversed by the brusatol treatment. Considering the nuclear accumulation of Nrf2 during Keap1- Nrf2∕ARE signaling pathway activation, we further determined the protein levels of Nrf2 in the nucleus. The result (Fig. 4B) showed that LBP treatment could significantly increase the level of Nrf2 in the nucleus compared with the LPS group (P<0.001), consistently, which was reversed by the brusatol treatment. 

###  Nrf2 inhibition by brusatol alleviates the antioxidant response induced by LBP 

**Figure 5:**
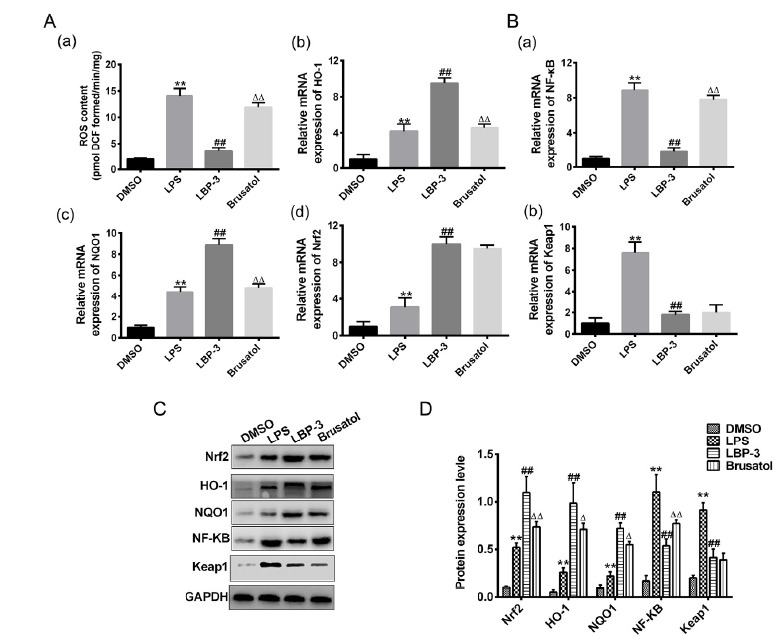
Nrf2 inhibition by brusatol alleviates the antioxidant response induced by LBP (**A**) Effect of brusatol on the antioxidant response in LPS induced septic kidney. (**B**) Effect of brusatol on the Keap 1- Nrf2∕ARE signaling pathway related genes. (**C**) Western blot analysis of antioxidant responses and Keap 1- Nrf2∕ARE signaling pathway related proteins. (**D**) Quantitative analysis of the Western blot results. **, P<0.01, *versus* the DMSO group; ##, P<0.01, *versus* the LPS group; ΔΔ, P<0.01, *versus* the LBP group. DMSO control group (DMSO): intraperitoneal injection with DMSO. LPS model group (LPS): intraperitoneal injection with LPS (5 mg/kg); LBP-3 group: giving intragastric administration with 800 mg/kg LBP 1h after LPS injection; Brusatol group: intraperitoneal injection with LPS together with 2 mg/kg of brusatol, then LBP was administrated 1h after.

 Moreover, the antioxidant responses induced by LBP after Nrf2 inhibition by brusatol were further investigated. As shown in Figure 5A the content of ROS in brusatol treated group increased significantly compared with the LBP-3 control. Consistently, the mRNA and protein expressions of HO-1, NQO1, Nrf2 after administration of brusatol were decreased (P<0.01; Fig. 5). Moreover, compared with the LBP-3 group, the gene expression levels of Nrf2 and Keap1 after brusatol treatment showed no significant difference when compared with the LBP-3 group. In addition, for the protein expression levels of Nrf2 and Keap1, we found that brusatol treatment has no significant effect on the Keap1 expression, while the Nrf2 expression was significantly inhibited (P<0.01, versus the LBP-3 group). All these results indicate that Nrf2 inhibition by brusatol can inhibit the Keap1- Nrf2∕ARE signaling pathway, and increase the expression of NF-κB, then further inhibits the antioxidant responses.

###  Nrf2 inhibition by brusatol reverses the protective effect of LBP on the LPS induced septic kidney injury 

**Figure 6:**
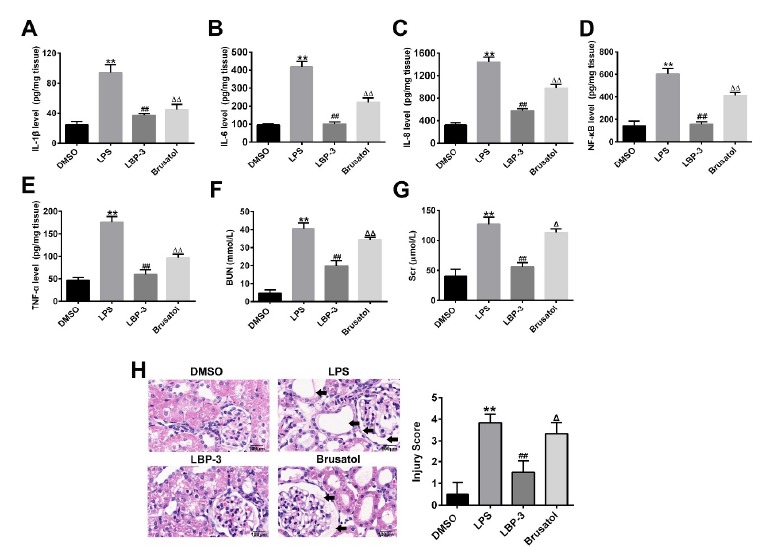
Nrf2 inhibition by brusatol reverses the protective effect of LBP on the LPS induced septic kidney injury. (**A-E**) Influence of brusatol on the expressions of immune factors in sepsis induced rat kidney. (**F**) Pathological morphology observation and scoring of kidney tissue among groups. **, P<0.01, *versus* the DMSO group; ##, P<0.01, *versus* the LPS group; ΔΔ, P<0.01, versus the LBP group. DMSO control group (DMSO): intraperitoneal injection with DMSO. LPS model group (LPS): intraperitoneal injection with LPS (5 mg/kg); LBP-3 group: giving intragastric administration with 800 mg/kg LBP 1h after LPS injection; Brusatol group: intraperitoneal injection with LPS together with 2 mg/kg of brusatol, then LBP was administrated 1h after.

 The ELISA results showed that the inflammatory cytokines (IL-1β, IL-6, IL-8, TNF-α and NF-κB) concentrations increased significantly after the treatment of brusatol, compared with the LBP-3 group (P<0.01; Fig. 6 A-E). Also, the concentration of BUN and creatinine raised dramatically after brusatol treatment (P<0.01, *versus* the LBP-3 group; Fig. 6 F,G), indicating that kidney function was declined by brusatol treatment. Consistently, compared with the LBP-3 group, HE staining showed that more inflammation cells aggregation and cellular swelling and infiltration were observed in the kidney tissue after brusatol treatment (Fig. 6H). The result indicates that Nrf2 inhibition by brusatol can reverse the protective effect of LBP on the LPS induced septic kidney injury.

## Discussion

 Inflammatory injury and oxidative-stress are important factors of the pathogenesis of sepsis and tissue damage^12^ ^,^ ^13^. Our study found that the concentration of IL-1β, IL-6, IL-8, TNF-α and NF-κB in rat kidney tissues increased significantly at 12h after injection with LPS, however, after different doses of LBP injection, the concentration of these inflammatory cytokines presented a significant reduction. These findings suggest that LBP treatment can play a protective role on the inflammation damage of septic rat kidney. 

 LBP has been shown efficient in diabetic kidney disease models; diabetic rats treated with LBP (10 mg/kg) for 8 weeks showed increased activity of antioxidant enzymes and increased scavenging of oxygen radicals^14^. Recently, Kawara et al. reported that oxidative damage caused by LPS induced AKI may be regulated through the Nrf2 signal pathway^10^. Nrf2 is a nuclear transcription factor which is related to oxidative stress reaction and plays an important role in cell defense and protection. Nrf2 mainly expresses in lung, kidney, small intestine and other organs, always accompanied by the occurrence of detoxification reaction^15^. Nrf2 regulates the expression of downstream genes that encode antioxidant protein and phase II detoxification enzyme through interaction with the Keap1 and antioxidant response element (ARE), thereby playing a role in cell protection. Nrf2-Keap1/ARE is an important signal pathway of oxidative stress system. In physiological state, Nrf2, combination with Keap1 in the cytoplasm, is in a relative inhibitory state and would not activate the downstream signal^14^. With oxidative stress stimulated by ROS and electronic, Nrf2 and Keap1 are dissociated and Nrf2 is transferred to the nucleus to combine with ARE, thus, promoting the transcription and expression of ARE downstream genes, such as antioxidant protein or pro-oxidant protein synthetase, to resist the destructive stimulus^15^. In this study, we found that the content of ROS, as well as the expressions of HO-1, NQO1, Nrf2 as molecular elements that respond to oxidative stress, in LPS group increased obviously compared with the control. More interestingly, the expression level of NF-κB and Keap1 in the Keap1-Nrf2∕ARE signaling pathway increased significantly after injection LPS. All these indicate that the oxidative stress stimulated by LPS could regulate the Nrf2-Keap1 dissociation and then promote the transcription and expression of the downstream antioxidant protein. With these, we further evaluated the role of LBP on the oxidative stress. After LBP treatment, the ROS content, as well as the expressions of NF-κB and Keap1, reduced significantly in a dose-depend manner. However, the expression levels of HO-1, NQO1 and Nrf2 were further increased, which indicates that LBP treatment can protect the septic kidney injury by activating the antioxidant responses through Keap 1-Nrf2∕ARE signaling pathway.

 Brusatol is isolated from the Brucea javanica plant. It has been shown to serve as an inhibitor of Nrf2 signaling^16^. The result of this study showed that brusatol treatment significantly increased the content of ROS, as well as the expression of NF-κB, compared with the LBP treated control. As expected, the expressions of HO-1, NQO1 after administration of brusatol were also decreased. Unexpectedly, the gene expression levels of Nrf2 and Keap1 after brusatol treatment showed no significant difference when compared with the LBP-3 group. For the protein expression levels of Nrf2 and Keap1, we found that brusatol treatment has no significant effect on the Keap1 expression, while the Nrf2 expression was significantly inhibited. This finding suggests that brusatol depletes Nrf2 protein through a post-transcriptional mechanism and through a Keap1-independent mechanism, which is consistent with the report by Olayanju *et al.* ^17^. All these results indicate that inhibiting Nrf2 can increase the expression of NF-κB, and further inhibit the antioxidant responses. Furthermore, a reversed effect on inflammatory cytokines concentrations was also observed compared with the LBP group. Complemented with the HE staining results, we found that Nrf2 inhibition by brusatol can reverse the protective effect of LBP on the LPS induced septic kidney injury.

## Conclusions

 Taken together, our results suggest that LBP can up-regulate the level of Nrf2 and down-regulate the pro-inflammatory cytokines, and further regulating the Keap1-Nrf2/ARE signaling pathway, consequently activating the antioxidant responses and reducing inflammation. Along with in-depth research of sepsis and inflammatory injury, the mechanism by which how to reduce the oxidative stress in sepsis damage to the body is still not very clear. Therefore, further studies and development are still needed, which could provide a better foundation for clinical treatment of septic kidney injury.
